# Untangling the immune basis of disease susceptibility

**DOI:** 10.7554/eLife.56886

**Published:** 2020-05-14

**Authors:** Ruy M Ribeiro, Luis Graca

**Affiliations:** 1 Laboratório de Biomatemática, Instituto de Saúde Ambiental, Faculdade de Medicina, Universidade de LisboaLisboaPortugal; 2 Instituto de Medicina Molecular, Faculdade de Medicina, Universidade de LisboaLisboaPortugal

**Keywords:** HLA, GWAS, CD8 T cell, natural killer cell, disease association, immunogenetics, Human

## Abstract

Interactions between immune cell receptors and proteins that determine disease susceptibility shed light on how different arms of the immune system are involved in three viral infections and Crohn's disease.

**Related research article** Debebe BJ, Boelen L, Lee JC, IAVI Protocol C Investigators, Thio CL, Astemborski J, Kirk G, Khakoo SI, Donfield SM, Goedert JJ, Asquith B. 2020. Identifying the immune interactions underlying HLA class I disease associations. *eLife* **9**:e54558. doi: 10.7554/eLife.54558

Why do people respond differently to the same infectious agent? A possible answer is that differences in the immune responses of individuals, caused by genetic variability between them, are responsible. Indeed, many studies have looked for associations between genes involved in immunity and disease outcome (Buniello et al., 2019), and it has been found that a gene complex called the human leukocyte antigen (HLA) system has a central role (Matzaraki et al., 2017).

The HLA genes encode the major histocompatibility complex (MHC). MHC proteins are found on the surface of cells and each one presents peptides from proteins within the cell (either proteins native to the cell or from foreign entities like bacteria) to immune cells. Thus, MHC proteins allow immune cells to recognize if a given cell is dangerous or not. For example, immune cells called CD8+ T lymphocytes recognize cells that have been infected with viruses because receptors on these T cells bind specific MHC proteins loaded with viral peptides on the surface of the infected cells. Likewise, other types of immune cells – such as natural killer cells, macrophages and dendritic cells – have receptors that bind to other parts of the MHC protein displayed by infected cells (Augusto and Petzl-Erler, 2015; Hudson and Allen, 2016).

The HLA is extremely diverse among individuals. It has been assumed that the association between some diseases and certain HLA alleles is evidence for a central role of CD8+ T cells in that disease. However, given that the HLA can also interact with receptors on other immune cells, disentangling the contributions of the different arms of the immune system remains a challenge. Now, in eLife, Becca Asquith of Imperial College and colleagues – including Bisrat Debebe as first author, researchers from various institutes in the UK and US, and the IAVI Protocol C Investigators – report the results of a new approach to disentangling these contributions (Debebe et al., 2020).

Debebe et al. reasoned that it may be possible to predict which immune cells are responsible for fighting a specific disease since receptors from particular immune cells interact with different regions of the MHC protein. For example, susceptibility to a disease may be associated with individuals carrying MHC proteins that are similar in the region that presents protein fragments to the T-cell receptor (which can be thought of as being similar in ‘T-cell receptor space’). If this is the case, then CD8+ T cells are likely involved in fighting the disease. The same reasoning can be made for associations with the other receptors, such as the killer immunoglobulin-like receptor (KIR) receptors in natural killer cells and leukocyte immunoglobulin-like receptor (LILR) in myeloid cells (Figure 1).

**Figure 1.**
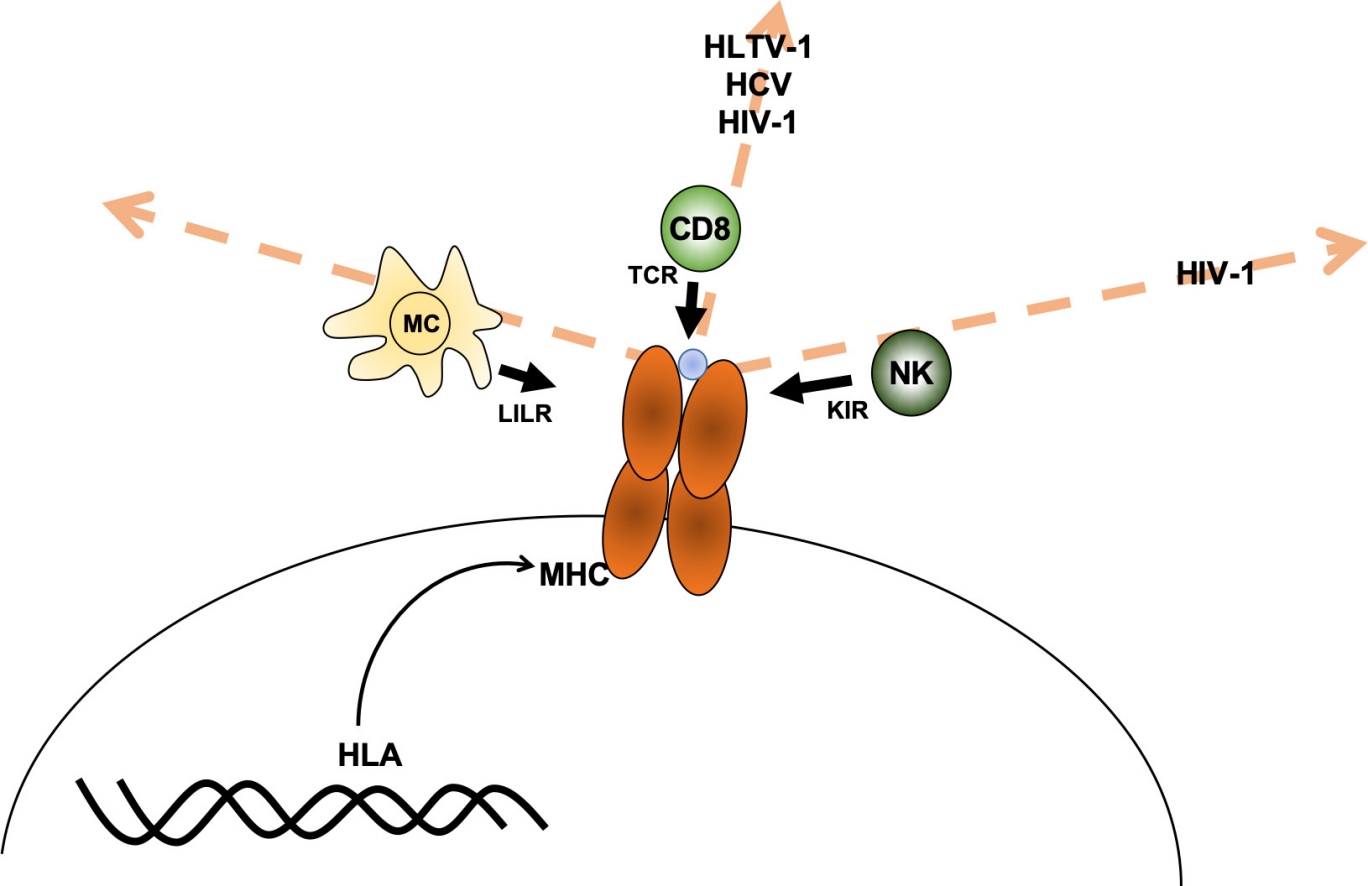
Axes of similarity in MHC proteins. Debebe et al. showed that similarities in regions of the MHC molecule that interact with different immune cell receptors can be thought of in terms of different 'spaces' (which are defined by an axis for each type of receptor). They found that MHCs that are similar in the T-cell receptor (TCR) axis are associated with disease outcomes for HLTV-1, hepatitis C virus (HCV) and HIV-1. Disease outcomes for HIV-1 are also associated with MHCs that show similarity in the killer immunoglobulin-like receptor (KIR) axis. For the diseases tested, no MHCs were found to be similar in the region that interacts with the leukocyte immunoglobulin-like receptor (LILR) carried by myeloid cells (MC). CD8: CD8+ T cells; NK: natural killer cells; blue circle: peptide.

The challenge in this method is defining MHC similarity. Debebe et al. did so by taking an HLA known to associate with a specific disease outcome and predicting its ability to bind to different immune cell receptors. Next, they compared this predicted binding ability to that of all other HLAs, and found the HLAs with the highest binding similarity. Finally, they checked if these HLAs are also associated with disease susceptibility.

Debebe et al. applied this method to three infections (human T-cell leukemia virus type one or HTLV-1; hepatitis C virus; and HIV-1) and one autoimmune disease (Crohn’s disease). They used well-characterized cohorts where both the HLA and the outcome of the disease were known for each individual.

They studied three known HLA-outcome associations for HTLV-1 and found that other HLAs that were similar in T-cell receptor space were also associated with HTLV-1. However, other HLAs that were similar to these three HLAs in LILR space or KIR space (see Figure 1) were not associated with HTLV-1. Thus, they concluded that HTLV-1 is most likely controlled by CD8+ T cells. They also studied three HLA-outcome associations for hepatitis C, reaching the same conclusion: disease outcome is mostly controlled by CD8+ T cells. For HIV-1, Debebe et al. found that natural killer cells and CD8+ T cells both have a role, which is consistent with previous results (Bashirova et al., 2011; McBrien et al., 2018). They also studied two known HLA-outcome associations for Crohn’s disease in a large sample (2650 individuals), but other HLAs that were similar in T-cell, LILR or KIR space did not have significant effect on Crohn's disease outcome.

The approach suggested by Debebe et al. generates insights into which immune cells may be involved in fighting a disease. There are two issues that need to be explored further. First, while we know a great deal about T-cell receptors, we know less about KIR, and even less about LILR binding (Hirayasu and Arase, 2015). This knowledge (or lack thereof) is part of the definition of similarity used by Debebe et al., which can and should be updated as more data becomes available (Gwozdowicz et al., 2019). The second issue pertains to the fact that it may be possible to define similarity in other ways, leading to different results that provide further insights into how genetic variability affects the immune response.
